# Osimertinib inhibits brain metastases and improves long-term survival in a patient with advanced squamous cell lung cancer: a case report and literatures review

**DOI:** 10.3389/fonc.2023.1188772

**Published:** 2023-09-14

**Authors:** Zhiqin Zhang, Jiamao Lin, Linke Yang, Yang Li

**Affiliations:** ^1^ Department of Radiation Oncology, Shandong Cancer Hospital Affiliated to Shandong First Medical University, Jinan, China; ^2^ Department of Radiation Oncology, Shandong Cancer Hospital and Institute, Shandong First Medical University, Academy of Medical Sciences, Jinan, China; ^3^ Department of Traditional Chinese and Western Medicine, Shandong Cancer Hospital and Institute, Shandong First Medical University, Academy of Medical Sciences, Jinan, China; ^4^ Department of Pathology, Shandong Cancer Hospital and Institute, Shandong First Medical University, Academy of Medical Sciences, Jinan, China; ^5^ Shandong Provincial Key Laboratory of Radiation Oncology, Shandong Cancer Hospital and Institute, Shandong First Medical University, Academy of Medical Sciences, Jinan, China

**Keywords:** non-small cell lung carcinoma, squamous cell carcinoma, EGFR mutation, targeted therapy, cancer

## Abstract

**Background:**

Squamous cell carcinoma (SCC) is one of the most common subtypes of non-small cell lung cancer, but its treatment options remain limited. Epidermal growth factor receptor (EGFR)–tyrosine kinase inhibitors (TKIs) have limited efficacy in the treatment of lung SCC. Here, we report an SCC patient who developed EGFR-T790M mutation and showed gefitinib resistance achieved an extremely long survival by taking Osimertinib alternatively.

**Case summary:**

A patient, 66-year-old non-smoking and drinking male with advanced SCC who was deemed inoperable at the time of diagnosis. The first genetic testing showed deletion mutation of exon 19 of EGFR. The patient was then treated with gefitinib with no significant efficacy. EGFR-T790M mutation was found in the second genetic test. The treatment regimen was changed to radiotherapy with Osimertinib, and the patient’s primary lesion and the brain metastases were well controlled.

**Conclusion:**

This typical case highlights the important role of Osimertinib in patients with SCC carrying EGFR mutations.

## Introduction

Lung cancer is the second most common cancer and the most common cause of cancer death worldwide. In 2020, 2.2 million new cases of lung cancer, accounting for 11.4% of the total 18 million cancer cases, were reported and 1.8 million new cancer deaths were related to lung cancer, accounting for 18% of all cancer deaths (1). The treatment of squamous non-small cell lung cancer, which constitutes 25%–30% of NSCLC, is challenging because of its specific clinicopathologic characteristics and rare incidence of targetable mutations (2). NSCLC has a poor prognosis, especially in stage IIIB/IV patients, with a 5-year overall survival (OS) rate of less than 5% (3).

The median survival time of patients with squamous cell carcinoma (SCC) was approximately 30% shorter than that of patients with other NSCLC subtypes (2). Here, we report a case of stage Ⅳ SCC patient who had lost the opportunity for surgery at the initial diagnosis. He was treated with first- and third-generation epidermal growth factor receptor (EGFR)–tyrosine kinase inhibitors (TKIs), and he is still alive over 5 years after treatment.

## Case description

**Figure 1:**
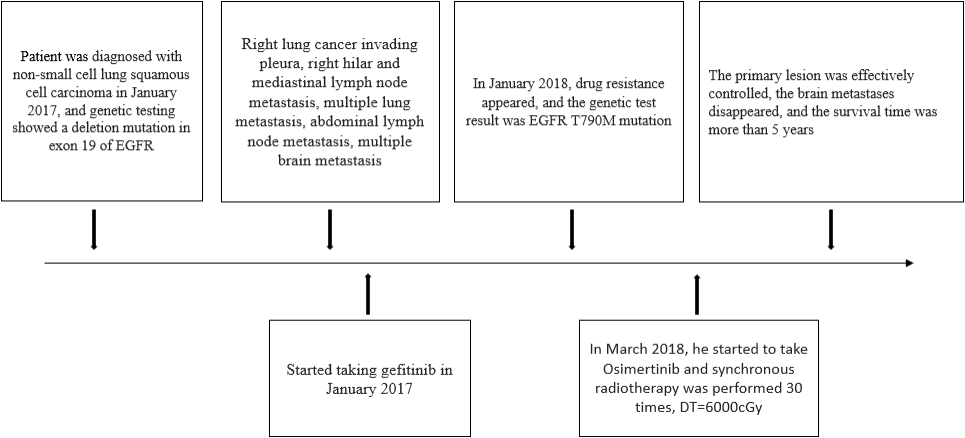
Timeline of our case’s treatment for advanced squamous cell carcinoma.

**Figure 2:**
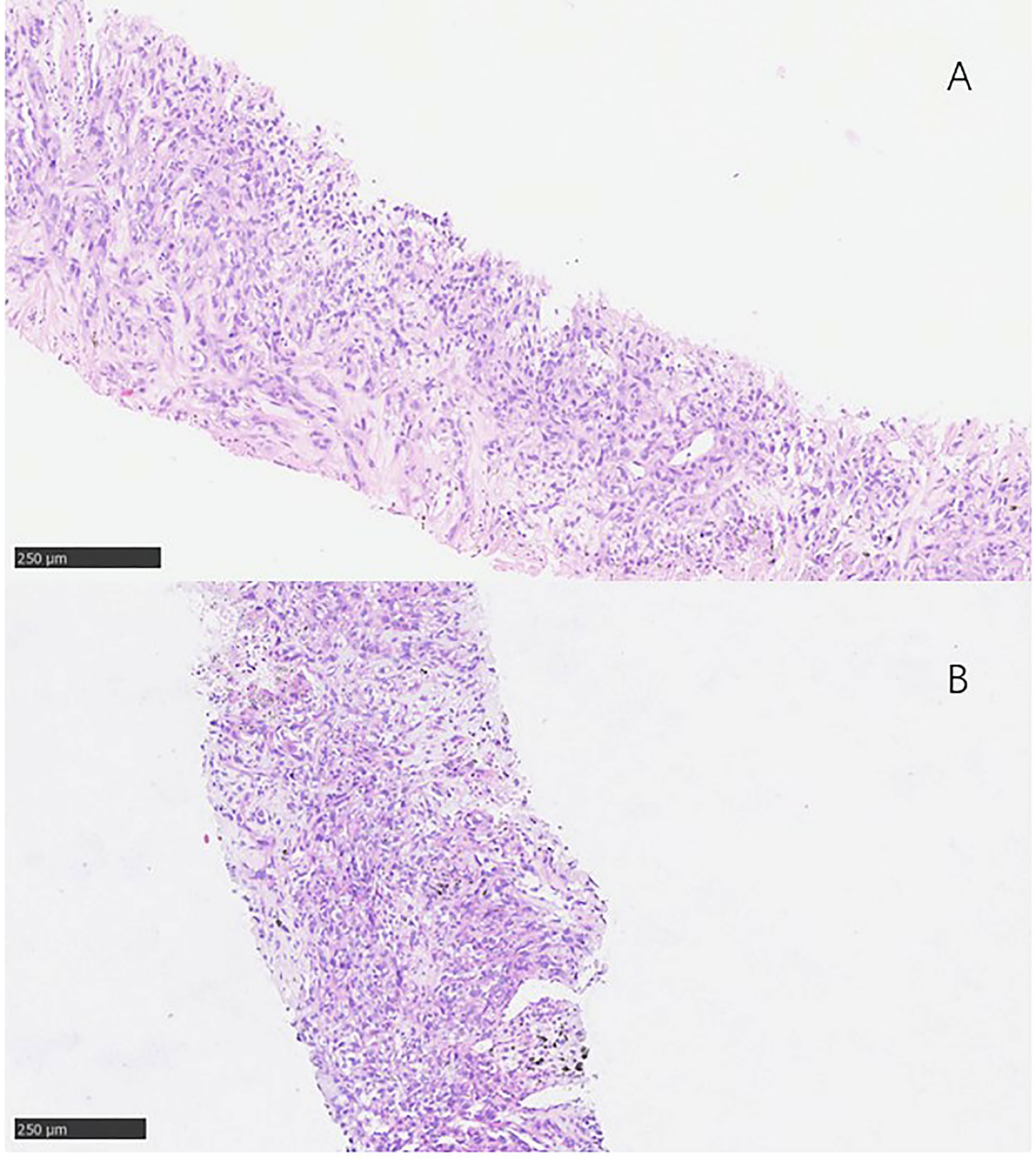
Pathological findings. **(A)** 3 January 2017, the pathological result was squamous cell carcinoma of non-small cell lung cance. **(B)** 17 January 2018, the pathological result was still lung squamous cell carcinoma.

**Figure 3:**
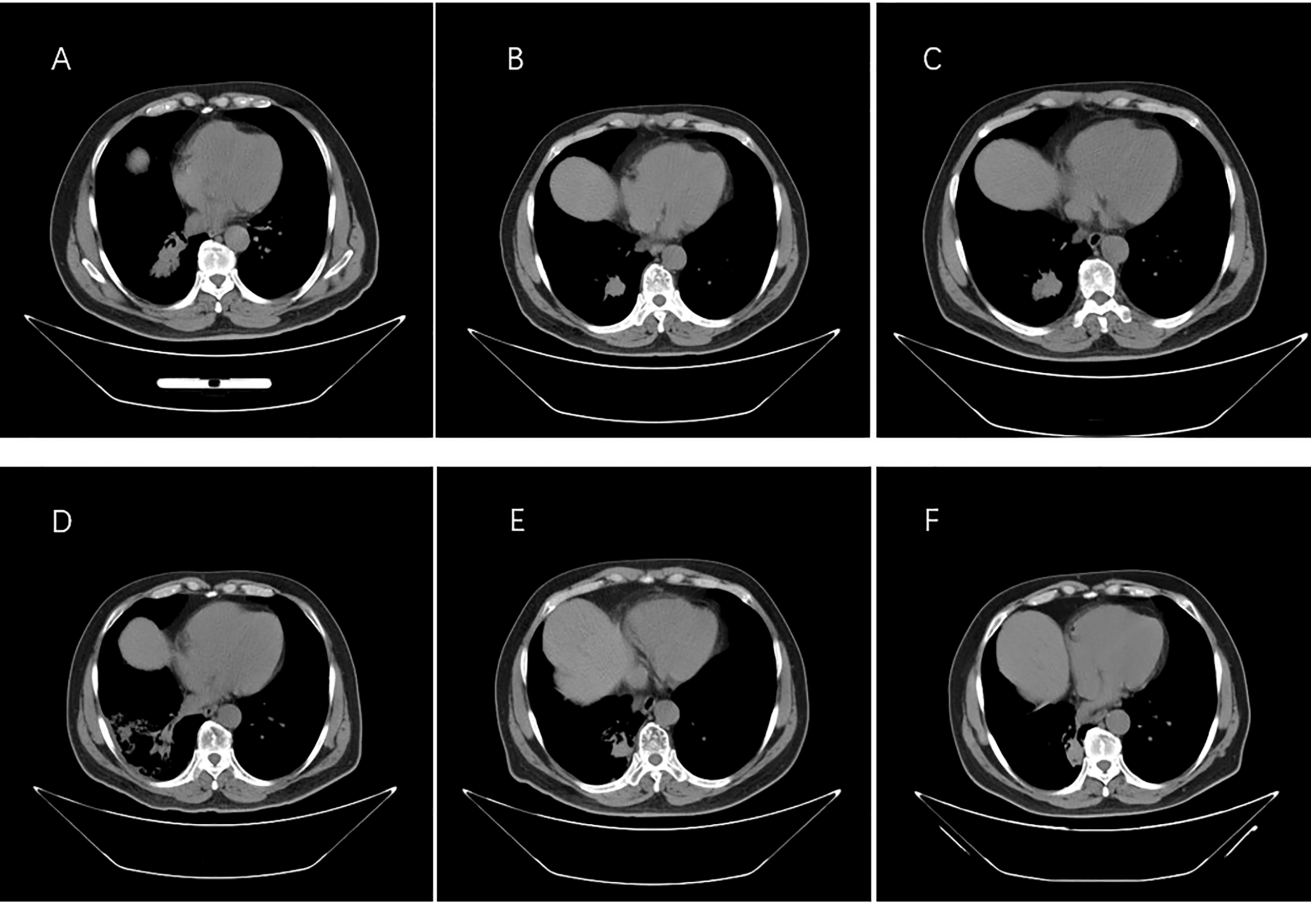
Images from computed tomography showing the tumor in the right lung in response to therapy. **(A)** Before therapy, imaging was performed on 30 December 2016. **(B)** Six months after taking gefitinib on 18 August 2017. **(C)** Resistance progresses on 20 January 2018. **(D)** After the radiotherapy on 5 June 2018. **(E)** One year after taking Osimertinib on 18 February 2019. **(F)** Last check on 20 May 2022.

**Figure 4:**
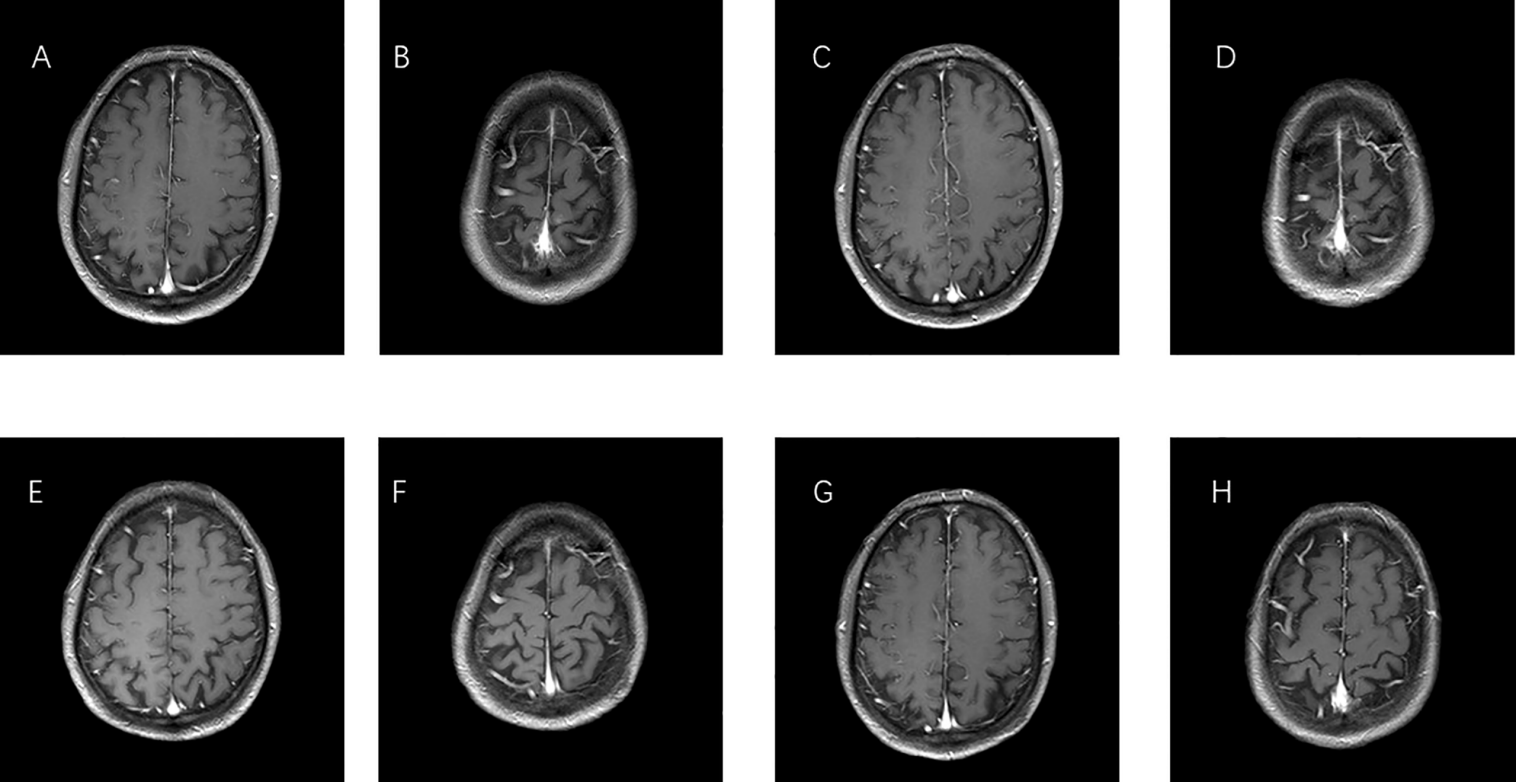
MRI images of brain metastases. **(A, B)** Before therapy, imaging performed on 30 December 2016. **(C, D)** One year after taking gefitinib on 19 March 2018. **(E, F)** One year after taking Osimertinib on 18 February 2019. **(G, H)** Last check on 20 May 2022.

On 29 December 2016, a 66-year-old Chinese male patient with no history of smoking and drinking presented to the Department of Oncology, Shandong Cancer Hospital and Institute (Jinan, China) with a cough and chest tightness for 1 month. Space occupying lesions in the lower lobe of the right lung were found during CT examination, and then he was admitted to our hospital for further treatment. At this time, the ECOG score was 1, and the patient had no previous history of related drugs and surgery. CT showed an irregular soft tissue mass in the lower lobe of the right lung with a cross section of about 4.8 cm × 4.6 cm, multiple lung masses, right hilar and mediastinal lymph node, and abdominal lymph node involvement, with metastases of right pleural, rib, and the brain. The clinical stage was T4N3M1, IV, and the pathological biopsy showed non-small cell lung cancer combined with immunohistochemical tendency of SCC. A deletion mutation of exon 19 of EGFR was found in genetic testing. The patient received 14 cycles of targeted therapy with gefitinib (250 mg/d, qd) from January 2017 to March 2018. The best response was stable disease (SD) (**Figure 1**). In March 2018, the CT reexamination showed the progress of the disease, and EGFR T790M mutation was found after genetic testing of specimen acquired by pathological puncture (**Figure 2**). Then the patient was admitted to the hospital for radiotherapy of the lower lobe of the right lung and metastatic lymph nodes, DT = 6000 cGy (200 cGy dose per time). In the same month, patient received targeted therapy with Osimertinib (80 mg/d, qd) until now. The effect was evaluated as SD. At this time, the patient’s clinical stage was T4N3M1, IV. After taking Osimertinib for more than 4 years, the primary lesion in the right lung was well controlled (**Figure 3**) and the brain metastases almost disappeared (**Figure 4**).

## Discussion

Here, we described a patient with stage IV advanced SCC. At initial diagnosis, the mutation of EGFR exon 19del was found. After taking gefitinib for more than 1 year, the patient developed drug resistance. The result of second genetic test showed EGFR-T790M mutation, which is an acquired drug resistance mutation. When treatment was switched to Osimertinib with the combination of primary and metastatic lymph node radiotherapy resulted in a long progression-free survival (PFS).

The first-generation EGFR-TKIs Gefitinib (ZD1839) was approved as first-line therapy for the treatment of patients with NSCLC harboring EGFR sensitive mutation, specifically. EGFR exon 19 deletions (ex19del) and/or EGFR L858R mutation in exon 21. These mutations occur in 10%–40% of patients with NSCLC. However, the patient’s condition deteriorated after 10–14 months of treatment with gefitinib. Studies have indicated that approximately 50% of the progression of NSCLC was due to the additional EGFR T790M resistance mutation (4–7). The patient was consistent with clinical cohort data during gefitinib use. During this process, the patient developed drug resistance.

In patients with non-adenocarcinoma (ADC) non-small cell lung cancer carrying EFGR mutations, clinical studies have shown that the median OS of patients treated with EGFR-TKIs is significantly higher than patients not treated with EGFR-TKIs, and there is no significant difference in clinical characteristics between patients who respond to EGFR-TKIs and those who do not (8). Based on the literature review, the efficacy of EGFR-TKIs in lung SCC with EGFR mutant is lower than that in adenocarcinoma. According to a clinical study, 33 (13.3%) of 249 patients with SCC included in the study had EGFR mutations. Twenty of these patients received EGFR-TKI (erlotinib or gefitinib) with a response rate of 25% (95% confidence interval, 8.7%–49.1%). PFS was 1.4 months, and OS was 14.6 months. Approximately one-third of patients with EGFR-mutated lung SCC have PFS of more than 6 months (9). EGFR-T790M is a common drug resistance mutation, resulting in about 60% of NSCLC patients with EFGR mutation who are resistant to EGFR-TKIs (10). There are two types of T790M mutations: primary mutation and acquired mutation. The acquired T790M mutation is usually the resistance gene generated after the first- or second-generation EGFR-TKI treatment. Both the primary and the acquired mutations showed good response to the third generation EFGR-TKI Osimertinib (11, 12). In NSCLC patients with acquired (Chiang, Huang et al., 2020) resistance to first- or second-generation EGFR-TKIs, Osimertinib is an alternative choice of treatment. Osimertinib was superior to platinum doublet chemotherapy with a higher rate (71% vs. 31%), a longer PFS (10.1 *vs.* 4.4 months) and median OS (26.8 *vs.* 22.5 months) (13, 14). Compared with traditional chemotherapy, EGFR-TKI–targeted therapy enable patients of NSCLC with EGFR mutations to achieve longer progression free survival and OS (15). In the patient who acquired the EGFR-T790M resistance mutation after taking gefitinib, we selected Osimertinib for the treatment that was successful.

Among the metastatic sites of advanced lung cancer, the central nervous system (CNS) is the most common site, and 20%–65% of patients will develop brain metastases during the course of the disease (16). In advanced lung cancer, 20%–65% of patients will develop brain metastases. Up to 50% of Asian patients with NSCLC carry EGFR-gene mutations. The cumulative incidence of brain metastases was significantly high in patients with EGFR mutations, with 46%, 64%, and 71% at 1, 3, and 5 years, respectively (17). Preclinical studies of Osimertinib demonstrated a more homogeneous distribution in the brain than other TKIs (18, 19). Osimertinib can delay the development of symptomatic CNS metastases. After taking Osimertinib for more than 3 years, the brain metastases even disappeared, which indicates that Osimertinib has a good therapeutic effect on metastasis.

At present, there are few studies on the efficacy of EGFR-TKIs for lung SCC. SCC only accounts for less than 1% in FLURA and AURA trials to explore the efficacy of Osimertinib in lung cancer. No clinical studies of patients with lung SCC carrying EGFR mutations have been performed yet.

In the case of newly diagnosed advanced lung cancer with brain metastases and bone metastases, the patient received gefitinib or Osimertinib combined with radiotherapy extended a survival time to more than 60 months. The disease was stabilized, the primary lesion was well controlled, the brain metastases disappeared, and no tertiary or quaternary adverse reactions occurred after Osimertinib treatment. The results suggest that Osimertinib might be the choice of treatment for patients of lung SCC with EGFR mutations.

## Conclusion

We present here a case with Osimertinib and radiotherapy treated advanced SCC. Throughout the course of treatment, patients showed significant responses to both Osimertinib and radiotherapy, with prolonged PFS and OS. The results suggest that Osimertinib might be a good choice for the treatment of patients with lung SCC accompanied by EGFR mutations.

## Data availability statement

The original contributions presented in the study are included in the article/supplementary material. Further inquiries can be directed to the corresponding author.

## Ethics statement

The studies involving human participants were reviewed and approved by Shandong Cancer Hospital. The patients/participants provided their written informed consent to participate in this study. Written informed consent was obtained from the individual(s) for the publication of any potentially identifiable images or data included in this article.

## Author contributions

ZZ is responsible for thesis writing and picture editing. JL was responsible for case data collection. LY is responsible for the production and scanning of pathological sections. YL is responsible for reviewing articles and revising formats. All authors contributed to the article and approved the submitted version.
